# “Ekbom syndrome: delirium engraved on the skin”

**DOI:** 10.1192/j.eurpsy.2024.1581

**Published:** 2024-08-27

**Authors:** C. Perez Aparicio, L. Rodriguez Rodriguez, M. J. Gordillo Montaño

**Affiliations:** Hospital Can Misses, Eivissa, Spain

## Abstract

**Introduction:**

Ekbom syndrome also known as Morgellons syndrome or delirium of parasitosis is a psychiatric condition where the patient has the absolute conviction of being infested in spite of medical evidence. Patients may even mutilate themselves or apply toxic substances in order to get rid of these hypothetical organisms. Sometimes they bring samples of these hypothetical parasites to the office to prove their existence, which is known as the “matchbox sign”, a pathognomonic finding.

**Objectives:**

The aim of this clinical case is to make visible the impact that this psychiatric condition can have on the patient’s quality of life

**Methods:**

We present the case of a 40-year-old woman from Peru who was admitted to the otorhinolaryngology unit for injuries compatible with necrosis of the right pinna. When the patient was examined, scars were found on the lower limbs and back. The patient justifies the scratching lesions with the presence of pathogenic organisms, with no trace of them by the physician.

**Results:**

The patient was evaluated by psychiatry service during her admission in otorhinolaryngology, being diagnosed with Ekbom’s delirium and starting treatment with 3 mL of Aripiprazole. Subsequently she was referred to the mental health unit where she left the follow-up until today.

**Conclusions:**

Different effective treatments have been described, among them pimozide, atypical antipsychotics and some SSRIs. However, the complexity of treatment arises when dealing with the irreducible idea that the patient has of being infested, refusing in most cases to receive psychiatric treatment. This can degenerate into major organic and psychological problems that turn the patient’s life into a real hell, which often end up losing much of their daily functionality. The fact of empathizing with the patient and trying to elaborate a plan adjusted to the reality and needs of the moment, can help us to establish a good therapeutic bond that facilitates an early start of treatment and greater therapeutic adherence, enabling a significant improvement in their quality of life.

**Disclosure of Interest:**

None Declared

